# Model systems in SDHx-related pheochromocytoma/paraganglioma

**DOI:** 10.1007/s10555-021-10009-z

**Published:** 2021-12-27

**Authors:** Krisztina Takács-Vellai, Zsolt Farkas, Fanni Ősz, Gordon W. Stewart

**Affiliations:** 1 Department of Biological Anthropology, Eötvös Loránd University, Budapest, Hungary; 2 Division of Medicine, University College London, Gower Street, London, WC1E 6BT UK

**Keywords:** Pheochromocytoma, Paraganglioma, Succinate dehydrogenase, SDH, Model organisms

## Abstract

Pheochromocytoma (PHEO) and paraganglioma (PGL) (together PPGL) are tumors with poor outcomes that arise from neuroendocrine cells in the adrenal gland, and sympathetic and parasympathetic ganglia outside the adrenal gland, respectively. Many follow germline mutations in genes coding for subunits of succinate dehydrogenase (SDH), a tetrameric enzyme in the tricarboxylic acid (TCA) cycle that both converts succinate to fumarate and participates in electron transport. Germline *SDH* subunit *B* (*SDHB*) mutations have a high metastatic potential. Herein, we review the spectrum of model organisms that have contributed hugely to our understanding of SDH dysfunction. In *Saccharomyces cerevisiae* (yeast), succinate accumulation inhibits alpha-ketoglutarate-dependent dioxygenase enzymes leading to DNA demethylation. In the worm *Caenorhabditis elegans*, mutated SDH creates developmental abnormalities, metabolic rewiring, an energy deficit and oxygen hypersensitivity (the latter is also found in *Drosophila melanogaster*). In the zebrafish *Danio rerio*, *sdhb* mutants display a shorter lifespan with defective energy metabolism. Recently, *SDHB*-deficient pheochromocytoma has been cultivated in xenografts and has generated cell lines, which can be traced back to a heterozygous *SDHB*-deficient rat. We propose that a combination of such models can be efficiently and effectively used in both pathophysiological studies and drug-screening projects in order to find novel strategies in PPGL treatment.

## Background

This review concerns the use of model systems in the study of a series of rare human diseases, known as pheochromocytoma/paraganglioma (PPGL), which have shed much light on the pathogenesis of neoplastic and metastatic disease in general [1]. As will be described, germline mutations in the genes coding for elements of the succinate dehydrogenase complex are important in this context, although the *SDHx* (denoting any one of *SDHA*, *SDHB*, *SDHC* or *SDHD*) genes are by no means the only offenders here. We will give a short overview of the basic biochemistry of the succinate dehydrogenase complex, followed by a history of the growth of understanding in the role of SDH in these tumors, followed in turn by a description and discussion of how different model systems have illuminated the basic pathophysiology and how these models might be used in the future.

### Basic biochemistry

**Fig. 1:**
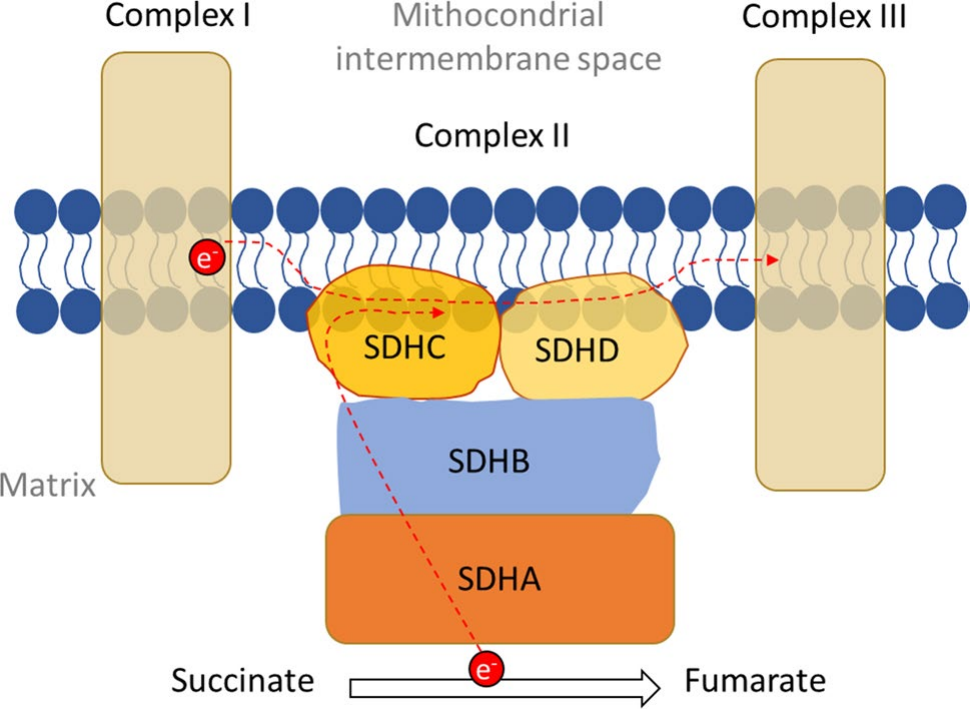
The structure of the four SDH subunits in the membrane. The SDH enzyme (also called complex II) is located in the mitochondrial inner membrane. SDHC and SDHD subunits anchor the enzyme to the membrane and act in electron transport; SDHA and SDHB subunits are responsible for succinate-to-fumarate conversion

This review will focus on mutations in succinate dehydrogenase subunits (collectively, SDHx) [2]. SDH is an established step in the tricarboxylic acid (TCA) cycle, converting succinate to fumarate with the evolution of FADH_2_ from FAD. Figure 1 shows a diagram of its structure. The enzyme is implanted in the mitochondrial inner membrane and also participates in electron transport. The enzyme consists of four subunits: SDHC and SDHD anchor the enzyme to the membrane and act in electron transport; SDHA and SDHB make up the biochemical enzyme. The hydrophilic flavoprotein SDHA hosts the majority of the catalytic cleft of the enzyme, and acts in concert with its hydrophilic partner SDHB. Subunit A binds FAD and possesses an active site to bind succinate. Subunit B coordinates three highly conserved iron–sulfur clusters (2Fe-2S, 4Fe-4S, 3Fe-4S) that undergo oxidation–reduction reactions as electrons pass. The two hydrophobic SDHC and SDHD partners serve as both membrane anchors and as the ubiquinone binding site [3].

In yeast, the four subunits of the SDH heterotetramer are known as Sdh1p-Sdh4p, where Sdh1p corresponds to SDHA, Sdh2p to SDHB, Sdh3p to SDHC and Sdh4p to SDHD. The four SDH assembly factors (SDHAF1-4 in the human) also follow this nomenclature derived from yeast: SDHAF1 (Sdh6p), SDHAF2 (Sdh5p), SDHAF3 (Sdh7p) and SDHAF4 (Sdh8p) (reviewed in Van Vranken et al., 2015: [4]).

The SDH assembly pathway has been elucidated recently and made it possible to identify factors which are required for the maturation of the two soluble subunits, SDHA and SDHB alone. After translation SDHA and SDHB are imported into the mitochondrial matrix as apo-proteins. SDHA is bound by assembly factor SDHF2/Sdh5p, which attaches the SDHA subunit to the cofactor FAD. Following flavinylation, Sdh5p is released and another subunit-specific chaperone, Sdh8p/SDHAF4, binds SDHA. As flavinylated SDHA is capable of oxidizing succinate, but binding of Sdh8p prevents the generation of superoxide, thus protecting the matrix from ROS [4].

Apo-SDHB also goes through a maturation process prior to final formation of the complex. First, insertion of the three Fe-S clusters occurs, then the assembly factors SDHAF1 (Sdh6p) and SDHAF3 (Sdh7p) associate with SDHB and act as chaperones to protect surface-exposed Fe-S clusters; next, Sdh6p and Sdh7p displace Sdh8p and facilitate SDHA-SDHB soluble dimer formation. At the end of the process, the SDHA-SDHB dimer is bound by the hydrophobic SDHC-SDHD subunits [4].

### Tumor formation

Pheochromocytoma and paraganglioma are tumors of the ‘neuroendocrine’ cells of the adrenal gland and sympathetic and parasympathetic ganglia, respectively. The tumors can be benign or metastatic. They are derived from secreting cells, and uncontrolled secretion of adrenaline-like catecholamines can be a clinical feature, with potentially dangerous physiological effects [1]. Our focus here is mainly on families carrying mutations in the genes coding for the four subunits of SDH.

The first case description of what is now known as pheochromocytoma is attributed to Sugrue in 1800 [5]. The term ‘pheochromocytoma’ was coined by the German pathologist Edgar Pick to convey the microscopic appearance of grey or dark coloured staining for chromium or silver salts, caused by a reaction between the stored adrenaline-type hormones within these cells and the chromium- or silver-containing fixatives. The existence of ‘paraganglioma’ was described in 1908 by Alezais and Peyron [6, 7]. These conditions are now regarded as fundamentally similar, the principal difference lying in their different anatomical localizations.

In the pathology, the classic appearance is of ‘zellballen’, nests of chromaffin cells interspersed in a fibrous stroma [1]. An Italian group has concluded that the tumors arise from a multi-potent stem cell, which develops into a disorganized vascular-plus-neural tumor, which closely resembles the cellular make-up of a hypertrophied carotid body under chronic hypoxic conditions [8, 9].

The fact that paragangliomas could be inherited was noted in 1933, in two Canadian sisters [10]. That mutations in the genes coding for subunits of the SDH enzyme were a major risk factor was discovered by mapping in 2000. Mutations in *SDHD* in PPGL were first shown by Baysal et al. [11]. Niemann and Muller showed that *SDHC* was involved [12], while the role of *SDHB* was demonstrated by Astuti and others [13]. Later, germline mutations in the assembly factor SDHAF2 were found [14]. It has recently been suggested that mutations in succinate ligase (*SUCLG2*), which attaches CoA to succinate in the TCA cycle, may also be a germline risk gene [15]. It was striking that the risk genes coded for a fundamental metabolic enzyme, rather than the replication-controlling enzymes that might be expected in a neoplastic condition [16].

While mutations in *SDHC* and *SDHD* subunit encoding genes typically predispose to benign tumors, *SDHA* and *SDHB* mutations are, for unknown reasons, strongly associated with high metastatic potential [17–19].

The complexity of the phenotype is illustrated by the fact that germline *SDHx* mutations may result in tumors other than PPGLs: *SDHB* and *SDHC* mutations may be responsible for gastrointestinal tumors (GIST) [20]. *SDHB* and *SDHD* mutations have also been reported to contribute to renal cell carcinoma [21] and papillary thyroid cancer [22].

In parallel with this work, germline mutations in many different genes predisposing to PPGL have been discovered. This has led to classification of PPGL into three groups, or’clusters’ in the parlance (see Table 1): 1,’pseudohypoxic’, the largest, which includes not only *SDHx* mutations but also other TCA cycle genes (e.g. *FH*) and genes coding for components of the’hypoxia inducible factor’ (HIF) system such as *VHL*, *PHD1*, *PHD2* and *HIF2A*; 2, the’kinases’ group; and 3, Wnt signalling [23–25]. Cluster 1 has been subdivided into 1a, TCA genes, and 1b, VHL-disease-related genes. We will not consider clusters 2 and 3 further here.

**Table 1:** Pathological classification of PPGL according to germline mutation and mRNA expression patterns. This table is based on the review by Crona et al. 2017 [26]

Cluster	Description	Fundamental germline or somatic mutations, if present	Transcriptome	Level of DNA methylation
1 a	Pseudohypoxia, TCA related	*SDHA*, *SDHB*, *SDHC*, *SDHD*, *SDHAF2*, *FH*	Increased HIF and downstream genes	High
1 b	Pseudohypoxia, HIF-system-related	*VHL*, *EPAS1*	Increased HIF and downstream genes	Intermediate
2	Wnt signaling	*CSDE1;* gene fusions affected *MAML3*	No increased HIF	Low
3	Kinase signaling	*RET*, *NF1*, *TMEM127*, *MAX*, *HRAS*	No increased HIF	Low

Although mutations or deletions in the genes coding for any of the four SDHx subunits give the same result (i.e. loss of SDH activity) there is an unexplained difference in phenotypes. There are differences in incidence (*SDHB* is commonest), the location of tumor (e.g. *SDHD* tumors are commoner in the head and neck, while in *SDHB*, the tumors are equally distributed between the head and neck and adrenal), tendency to metastasise (common in *SDHA* and *SDHB*), penetrance (greatest in *SDHC,* least in *SDHB*) [25].

The tumors arise via a classic ‘two-hit’ mechanism in which the wild type allele is somehow disabled, either by deletion or by mutation. The loss of the wild-type in the affected cells can be seen on DNA analysis and on histochemistry [27, 28].

**Fig. 2:**
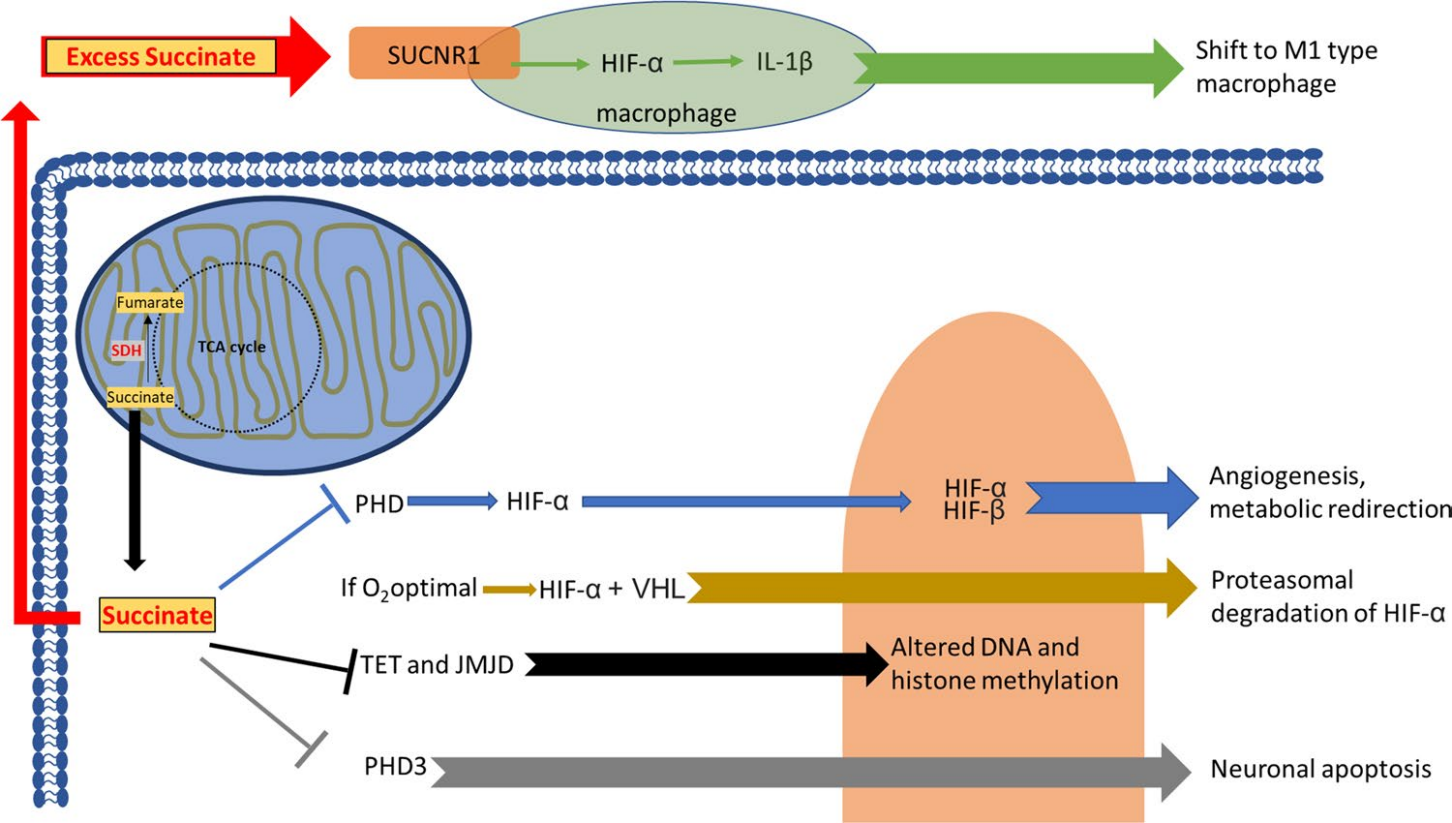
Succinate signaling pathways in the cell. SDH, a key enzyme of the TCA cycle, converts succinate to fumarate in the mitochondria. However, when this conversion is incomplete, excess succinate influences different signaling pathways in the cell. Succinate inhibits a number of alpha-KG-dependent dioxygenases: (1) prolyl-hydroxylases (PHD) are key players in the hypoxia-response system: under normoxic conditions PHD hydroxylates HIFα, which is ubiquitinated by VHL and degraded in the proteasome. However, succinate, by mimicking a pseudohypoxic environment, inhibits PHD and HIFα becomes active. (2) Succinate inhibits JMJD family KDMs and the TET family of 5mC hydroxylases, which causes alterations in histone and DNA methylation patterns. (3) Neuronal apoptosis can be affected by abnormal activity in PHD3, another alpha-KG-dependent dioxygenase when inhibited by succinate. Extracellular succinate is a ligand of SUCNR1, a GPCR, which is present on the surface of many human cell types. Succinate is also an inflammatory signal in the tumor microenvironment by inducing IL-1β expression through HIF-1α, which can lead to a switch of M1 type macrophages

The mechanisms by which loss of SDH activity seems to lead to neoplastic change in the pseudohypoxic group are illustrated in Fig. 2.

The first result is a primary loss of the biochemical activity of the enzyme, which blocks the TCA cycle, depriving the cell of a major ATP source and causing a build-up of succinate. The cell must then generate ATP from glycolysis, principally, mimicking the ‘Warburg effect’ that is seen in a broad range of cancer cells. If the cell can survive on glycolysis, then this switch in metabolism may not be a bad thing, since it provides carbon for anaplerosis, notably by switching on pyruvate carboxylase, which unites pyruvate with CO_2_ to form oxaloacetate, a potential feed molecule [29].

The secondary effect on mitochondrial dysfunction is an excess reactive oxygen species, a classic byproduct in SDH deficiency, creating carcinogenic effects [30].

One of the fundamental effects is that SDH deficiency, which causes an upstream build-up of unmetabolised succinate, results in inhibition by succinate in a series of enzymes known as α-KG-dependent (alpha-ketoglutarate-dependent) dioxygenases [31, 32]. There are about two dozen of these enzymes in human biochemistry, with a series of different functions. In each case, there is a core reaction in which one O_2_ molecule is split. One oxygen atom is transferred to α-KG- forming (by oxidative decarboxylation) the product succinate, while the other oxygen is transferred to another substrate with its own product. In any kind of SDH deficiency, the local accumulation of succinate can inhibit, not only by product inhibition but also by competitive substrate inhibition, any local α-KG-dependent dioxygenases [33]. Thus, one enzyme deficiency creates multiple regulatory defects across metabolism and the genome.

This ‘one-to-many’ relationship may be very important for PPGL because that two of the human α-KG-dependent dioxygenases are key players in the hypoxia-response system. This pair are proline hydroxylases 1 and 2 (PHD1, PHD2). In the HIF system, the transcription factor HIF is synthesized at a constant rate. The mature HIF is acted on by one of these prolyl hydroxylases, and *the rate of the reaction depends on the local oxygen concentration*. If the reaction is fast, HIF hydroxylation is complete, and if so, HIF is ubiquitinated by the VHL protein, and destroyed in the proteasome. Consequently, there is no active HIF. If oxygen is short, the prolyl hydroxylase enzyme slows down, HIF does not get hydroxylated, and VHL does not ubiquinate the transcription factor: HIF survives (‘is stabilised’, in the parlance) and the oxygen sensor system, which is very powerful, is activated [31].

This is how these tumors are regarded as ‘pseudohypoxic’. The mutation leads to a situation in which the cell behaves as if the oxygen tension is very low whereas in reality it is not. There is constitutive activation of the HIF system. This parallels the situation in von-Hippel-Lindau disease, in which there are mutations in the *VHL* gene coding for the ubiquitin ligase known as VHL, again leading to constitutive activation of the HIF system. The clinical spectrum is different, in that haemangiomas are the most prominent abnormality, but pheochromocytomas can occur, albeit less commonly [34].

There are many other human α-KG-dependent dioxygenases [35]. A number act as DNA demethylases, while others act as histone demethylators. Tonic inhibition can lead to a ‘hypermethylator’ epigenetic state in which transcription of many genes can be suppressed [32, 36, 37].

Another idea is that a ‘neuronal apoptosis’ (programmed cell death in neurons) can be affected by abnormal activity in PHD3, another α-KG-dependent dioxygenase which is inhibited by accumulating succinate. PHD3 (aka EGLN3) is required for apoptosis after the withdrawal of the growth signaler NGF (neuronal growth factor) [38].

Further, surplus succinate secreted by cancer cells can activate the HIF system in tumor-associated macrophages via a PI3K signaling receptor [39]. Succinate also has other signaling roles [40].

Another effect exerted by secreted succinate on PPGL tumorigenesis is the activation of GPR91, a succinate receptor also known as SUCRN1. SUCRN1 is a G-protein-coupled receptor, which was identified more than a decade ago as the cognate receptor of succinate [41]. Recent data show that GPR91 is upregulated if SDHB is silenced. GPR91 induction can be also observed in *VHL*-mutant renal clear cell carcinoma cells, and also in HIF2alpha-overexpressing HepG2 cells, raising the possibility that SUCNR1 itself might be one of the many targets of increased transcription which follows activation of the HIF system [16, 42]. In ischemic retina, activation of GPR91 by succinate in retinal ganglion cells resulted in release of pro-angiogenic factors such as VEGF and angiopoietins Ang-1 and Ang-2 in a HIF1α-independent manner [43]. Similarly, in human umbilical vascular endothelial cells, SUCNR1 activation led to upregulation of VEGF expression through activation of signal transducer and activator of transcription 3 (STAT3) and extracellular regulated kinase (ERK)1/2 [44]. These findings suggest that PPGL tumor cells—through accumulation of succinate—might stimulate proliferation of neighboring vascular endothelial cells by a paracrine signaling mechanism [45]. Whether the zellballen tumor architecture (see above) is affected by this process remains unknown, but recent data indicate that abnormally accumulated succinate in the tumor microenvironment also influences processes of inflammation. Succinate induces production of a key inflammatory cytokine interleukin-1β (IL-1β) through HIF-1α [46]. On the one hand, accumulation of succinate in the microenvironment can enhance tumor-associated inflammation, which leads to the release of bioactive molecules (e.g. proangiogenic factors, growth factors, extracellular matrix-modifying enzymes) that favor cancer cell growth, survival and tumor progression [47]. On the other hand, activation of IL-1β in the tumor microenvironment can induce shift of macrophages into the M1 subtype [48], thus contributing to host defense mechanisms and anti-tumor immunity [49].

**Fig. 3:**
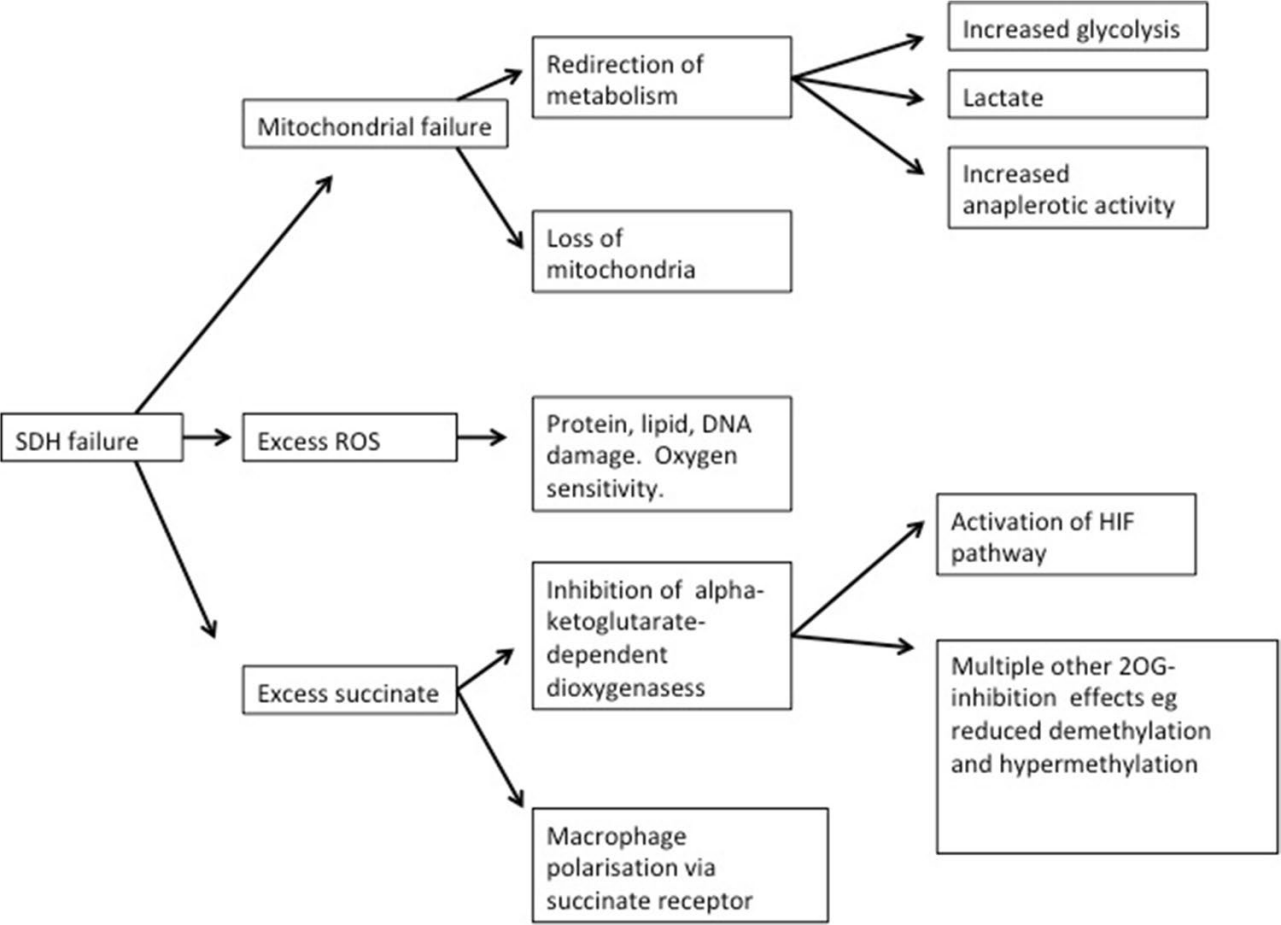
Multiple mechanisms of tumorigenesis in SDH deficiency. First, there is failure of the mitochondrion and oxidative phosphorylation. There is a redirection of metabolism towards glycolysis, now the main provider of ATP, with possible production of lactate but also redirection of pyruvate (via pyruvate carboxylase) into anaplerotic (i.e. synthetic) pathways which are thought to be helpful in tumor growth. Second, the faulty SDH leads to excessive reactive oxygen species production, which, if it exceeds the scavenging powers of the cell, can lead to damage to proteins, DNA and lipids. Third, the excess succinate building upstream of the metabolic block can inhibit α-KG-dependent dioxygenases, with multiple possible consequences, especially the activation of the HIF pathway and the inhibition of demethylase enzymes, disturbing epigenetic regulation in many possible ways. Fourthly, excess succinate can activate the G-protein-coupled succinate receptor, SUCNR1, with further multiple possible effects

So, by these multiple pathogenic pathways, summarized in Fig. 3, it can be seen how a mutation in a TCA gene can lead to cancer. In particular, the potential consequences of inhibition of α-KG-dependent dioxygenases are both legion and very far-reaching. It is not simply a matter of defective energy supply. The discovery that mutations in metabolic genes such as SDHx could be fundamental to these familial cancers prompted studies of metabolism in other cancers, giving multiple new insights into cancer generally. This is now a major topic [50].

One way to investigate these questions is with model animal systems, which we will now go into.

## Model systems

### Pathophysiology

When we consider the above pathogenic mechanisms, then it is natural to think of ways of testing them out experimentally. Biologists have spent much time working on ‘model organisms’ which can be studied in the laboratory. This approach can work because the basics of life (including SDH) are common between humans and the most primitive and simple organisms. Many of these model methods have been applied to this problem. So far most of the studies pointed out that there is no sufficient animal model to test SDH functions. More suitable *in vivo* models are needed to unravel underlying pathogenic mechanisms and to develop therapeutic strategies. Emerging models in different organisms have been developed that we will summarize below. Both invertebrate and vertebrate models have been used to investigate and characterize SDHx functions.

Table 2 summarizes the advantages and limitations of the various models used for PPGL research.

**Table 2:** Comparisons of different model systems

Organism/system	Percent of genome shared with human	Percent of SDH complex identity with human	Advantages in terms of PPGL	Limitations in terms of PPGL
Yeast	40%	47%	Haploid *SDHB* KO is viable	Unicellular
Nematode/worm	52%	52%	Metabolically, very similar to human; can be used for large scale drug screens	Has only a few neuroendocrine cells. Does not develop tumors
Fruit fly	60%	53%	Metabolically, very similar to human; can be used for large scale drug screens	Germline *SDHx*-mutated do not develop tumors
Zebrafish	70%	77%	Similar metabolic consequences to human and nematode	Germline *SDHx*-mutated do not develop tumors
Mouse	92%	90%	Applicable xenograft models	Germline *SDHx*-mutated do not develop tumors
Rat	92%	89%	Can develop PPGL tumors with germline *sdhb* mutations; RS0: best xenograft model to date	To date, poorly investigated
Dog	94%	88%	Spontaneously develops PPGL tumors, with similar germline risk genes	Expensive to maintain dog colonies; but possible to exploit routine domestic dog veterinary cases

We will consider the models in ascending order of laboratory complexity. First, unicellular systems (cultured cells, yeast), then invertebrates (the nematode *Caenorhabditis elegans*, the fruit fly), then non-mammalian vertebrates (Zebrafish), finally, mammals (mouse, rat and dog).

2.1.1 *Cultured cells*. Cultured cells, immortalised in some way, have a major role in biological research, and the study of SDHx mutations in PPGL has been no exception.

Generation of cell lines derived from PPGL-type tumors has presented multiple challenges, partly because of the histological complexity of these tumors, but also because pheochromocytomas and paragangliomas occur relative rarely spontaneously in rodents [51], although they can be induced chemically or by irradiation in mice and rats (reviewed most recently by Bayley and Devilee 2020 [52]).

Table 3 lists the cell lines which have been used in PPGL research related to *SDH*x genes. Section 1 of the table lists cell lines has germline *SDHx* modifications and which can be considered authentically ‘Cluster 1 pheochromocytoma-like’. These have been the most difficult to make. The Italian team immortalised cells from an *SDHC* tumor and an *SDHD*, using hTERT and/or SV40 large tumor antigen [53] and have exploited these cells extensively, increasing understanding of the pathological nature of PPGL tumors [9, 54]. The French team made immortalized cells from a mouse bearing a ‘floxed’ *Sdhb* gene [36] and these too have been investigated in depth, especially in the field of epigenetics, to demonstrate the ‘hypermethylator’ phenotype [55]. They were also used to study metabolism [56]. The exact nature of these cells has been questioned [52], although the epigenetic observations are undoubtedly very cogent to PPGL.

**Table 3:** Model systems: cultured cells. The table is in 3 sections: (i) pheo lines with SDHx knocked out, (ii) pheo lines with or without SDHx silencing, and (iii) non-pheo lines that have been used

System	Species	Nature	Biological character	Known genetic make-up	Metabolic features	Lessons Learnt	Reference(s)
*Pheo or pheo-like lines with germline SDHx knocked out or mutated*							
PTJ64i PTJ86i	Human	Immortalized cell lines derived from *SDHC*- and *SDHD*-mutated human patients. Immortalised with retroviral-mediated transduction with hTERT and/or simian virus 40 large tumor antigen	On flow cytometry, showed ‘immature mesenchymal, hypoxic, vasculoneural markers’ similar to tumors	Derived from *SDHC* c.43C > T (p.Arg15*) and *SDHD* c.27delC (p.Val10Phefs*5) human tumors	-	Importance of NOTCH signaling [53]. Importance of miR-200a,b,c and miR-34. Effects of PPAR-alpha inhibition [54]	[9, 53, 54]
imCC	Mouse	Adrenal cells derived from mice bearing a ‘floxed’ *SDHB* exon 2	Doubt as to whether these are true ‘chromaffin cells’. See [52]	Deletion of *SDHB* exon 2	Excess succinate, depleted fumarate	Hypermethylation phenotype	[36, 55, 56]
RSO; RS1/2	Rat	*Sdhb* +/- rats were irradiated and some developed pheochromocytomas. Tissue from tumors was injected into NSG mice to give xenografts. Cells from these were incubated in hypoxic media and two lines were prepared: RS0 (*sdhb*-/-) and RS1/2 (*sdhb* +/-)	Convincingly ‘chromaffin’	*sdhb*-/- (RS0) sdhb +/- (RS1/2)	Sensitivity to oxygen; excess succinate and lactate; high dopamine	Successfully used in mouse xenografts	[57]
*Phaeochromocytoma lines with or without SDHx silencing or KO*							
PC12	Rat	Derived from a rat pheochromocytoma and passaged in further rats	Classic neuronal cells. In NGF, will differentiate into neuron-type cells. Contain chromaffin granules and many characteristic catecholamine-related enzymes	Mutation in *Max,* gene coding for Myc partner protein Max [58]	Expresses many enzymes of catecholamine metabolism	Innumerable studies in general neuroscience	[59]
MPC 4/30	Mouse	Pheochromocytoma cell line derived from irradiated *Nf1* KO heterozygote mouse	Show chromosomal instability	Derived from mouse with heterozygous knockout of neurofibromatosis gene *Nf1*	Not SDH-deficient, but *sdhb* was knocked down by one group [60]	sdhb knock-down used to investigate expression of iron-transport proteins [60]	[61] [60]
MTT cells ‘mouse tumor cells’	Mouse	More aggressive derivative of MPC	Pheo-like	Ultimately derived from mouse with heterozygous knockout of neurofibromatosis gene *Nf1.* No *Sdhx* variation	Catecholamine secreting	Later used in allograft [62]	[63]
hPheo1	Human	Human pheo-derived cell line. Sporadic case, no known germline risk gene present. Immortalised with lentivirus-h TERT	Express chromogranin, COMT, TH	Deletion at 16p (does not affect SDHA,B,C,D)	Do not synthesise catecholmines		[64]
N2a, *sdhb* silenced	Mouse	Neuroblastoma (pheo-like) N2a silenced in *Sdhb* (Non *sdhb-*silenced MPC and MTT also studied)	Neuron-like	*SDHB* KD	SDHB very much reduced on western blots	Effect of piperlongumine	[65]
PC12 with *sdhb* knockdown	Rat	Modified PC12 cell line		*Sdhb* knockdown	Catecholamine oversecretion	Knockdown of *SDHB* led to increased catecholamine synthesis, increased ROS, and HIF stabilization	[66]
MTT, *sdhb* silenced	Mouse	Mouse pheo line		*Nf1* +/-	60% reduction in SDH activity; increased succinate	Effect of fibroblast co-culture	[67]
hPheo1 *SDHB* KD	hpheo1 shRNA *SDHB*	SDHB knock-down of hPheo1 cells	ditto	*SDHB* -/-	Increased succinate, decreased SDH on western blots	Used to study polyamine pathway and its inhibition	[68]
*Non-neuroendocrine cell lines that have been used, for gene knock-out, gene silencing or pharmacological inhibition of SDHx*							
SDHC E69	Mouse	Mouse fibroblasts. A copy of the *C elegans* point mutation in *sdhc* known as *mev*-1, which reduces but does not abolish, sdhc activity, and causes generation of ROS	A fibroblast and not an adrenal or neuroendocrine cell	*sdhc* point mutant p.V69 > E	Evidence of increased ROS	Sensitivity to oxygen; increased superoxide; increased apoptosis	[69]
HEK293 human embryonic fibroblasts	Human	Human embryonic kidney line. *SDHD* knockdown	Fibroblast	*SDHD* KD	SDH activity reduced 50%	To study effect of SDH silencing on HIF-alpha prolyl hydroxylase	[31]
Hep3B HeLa	Human	Human hepatoma and cervical cells, *SDHB* silenced	Non-pheo	*SDHB* KD	SDH much reduced on western blotting	Increased HIF-1alpha. Major microarray mRNA expression changes consistent with tumor measurements	[42]
HEK293 Hep3B	Human	Human embryonic kidney and hepatoma cell lines. SDH inhibited by 2-thenoyltrifluoroacetone (TTFA); *Sdhd* was silenced by siRNA	Non-pheo	*SDHD* KD	SDH much reduced on western blotting	Epigenetic hypermethylator changes	[70]
HEK293	Human	Human embryonic kidney line. *SDHB* knockdown	Non-pheo	*SDHB* KD	Diminished SDH on western blots	Effects of hypoxia on alpha ketoglutarate dependent dioxygenases	[71]
iMEFs sdhb -/- (irradiated mouse embryonic fibroblasts)	Mouse	Immortalised mouse cells with *SDHB* KO. Prepared from a conditionally knocked-out *Sdhc* mouse	Non-pheo	*SDHB -/-*	Diminished SDH on western blots; increased succinate, decreased fumarate; decreased SDH activity	ditto	[71]
iMK Sdhb -/- (immortalized mouse kidney)	Mouse	ditto	ditto	ditto	ditto	ditto	[71]
HCT116 SDHB KO 7, 23	Human	Colo-rectal cancer cells, *SDHB* removed by CRISPR	Non-pheo	*SDHB* -/-	Accumulated succinate, decreased fumarate	Very comprehensive metabolomics survey. Dependence on glutaminolysis. Susceptibility to BET inhibitors	[72]

More recently, the Tischler-Powers American team, in a major *tour de force* of experimental technique, have prepared two rat-derived cell lines containing *sdhb* deletions: ‘RSO’ is *sdhb-/-*, while ‘RS1/2’ is *sdhb* +/- [57]. A series of steps starting with a floxed *sdhb* rat and running through a mouse xenograft stage followed by novel cell cultures in hypoxic and low-serum conditions allowed the isolation of these cells, which seem set to provide an important resource.

In Section 3 of the table, there are listed pheo-like cell lines in which *SDHx* mutations are not fundamental. The classic is the PC12, a line derived from a spontaneous rat tumor of (as we understand it) unknown precise genomic composition, although it is known to contain a mutation in *Max*, encoding a partner protein for Myc [58]. (*Max* is a risk gene for human PPGL in Cluster 3[25].) PC12 cells display dense chromaffin-like granules and synthesize catecholamine neurotransmitters dopamine and norepinephrine. This line has been used extensively in general neuronal research as well as in PPGL research, where *sdhb* has been knocked down by RNA silencing, e.g. to study catecholamine metabolism [66]. The heterozygous *Nf1* +/-mouse [73] provided the ‘MPC’ (mouse pheochromocytoma) line, derived from a pheochromocytoma in the mouse [61]. *Sdhb* has been knocked down in this line [60], to study the potential therapeutic effect of ascorbic acid, a key cofactor for α-KG-dependent dioxygenases [32]. The ‘MTT’ (mouse tumor cells) line was derived from MPC and is more metastatically aggressive [63]. This too has had its *sdhb* silenced to study growth and invasiveness under *sdhb* knockdown [67]. Silencing led to a 60% reduction in SDHB expression, and it was observed that the *SDHB*-silenced MTT cells took up lactate secreted by fibroblasts when they were co-cultured with them in a monolayer. Lactate produced by cancer-associated fibroblasts was also taken up by pheochromocytoma spheroids when a 3D-co-culture was performed and *SDHB* was silenced. In addition, in the latter case the migratory potential of spheroids became increased, as a result of *SDHB* silencing.

The ‘hPheo1’ (human pheo 1) was derived from a sporadic human pheochromocytoma of unknown genomic landscape, using lentivirus h-TERT immortalization [64]. This too has been *sdhb* silenced, to study the effect of polyamine compounds [68].

In the third part of Table 3, Non-chromaffin cell lines that have been used for gene knockout, gene silencing or pharmacological inhibition of SDHx’, there are listed a series of non-chromaffin cells that have been employed in SDHx research. A number have been produced from conditional knockout *sdhx* mice, including iMEF *sdhb*-/- (immortalized mouse embryonic fibroblasts) and iMK *sdhb*-/- [71], and MEF and BMK (baby mouse kidney) by the Spanish team [74, 75]. The Japanese team mimicked the *C elegans mev-1* oxygen-sensitive point mutation in mouse fibroblasts, introducing to these cells the pV69 > E mutation in *sdhc* [69]. One of the key advances in the understanding of PPGL pathophysiology (that an alpha-ketoglurate-dependent dioxygenase could be inhibited by excess succinate) was made in the HEK293 (human embryonic fibroblast) line, subjected to *SDHB* knockdown, by Selak and others [31].

Small-molecule SDH inhibition has been used in HeLa cells and H295R adrenocortical cells [76]. The SDH inhibitors itaconate and atpenin, or siRNA treatment specific for *SDHB*, did not negatively affect chromaffin cells, in contrast to HeLa or H295R cells. After SDH inhibition or silencing, chromaffin-type PC12 cells showed increased expression of glutaminase-1 (GLS-1). BPTES, a GLS-1 inhibitor was able significantly to inhibit proliferation of SDH-defective PC12 cells [76]. This is potentially important and may suggest a mechanism by which SDH germline mutations confer susceptibility to neuroendocrine tumors: that chromaffin-type cells may be able to survive knock-out of both SDH alleles due to their ability to use glutamine as an anaplerotic substrate.

Kitazawa and others deleted *SDHB* in HCT116 cells (of colorectal cancer origin) by CRISPR to make an effective drug-screening survey [72].

2.1.2 *Saccharomyces cerevisiae*, or yeast, has contributed significantly to understanding the molecular pathogenesis of tumorigenesis resulting from defective SDH [77]. Yeast studies have shed much light on one of the proposed pathophysiological mechanisms, inhibition of DNA demethylation [37].

Table 4 shows yeast, and worm, fruit fly and zebra fish models.

**Table 4:** Model systems in SDHx PPGL: yeast *S. cerevisiae*, worm, *C. elegans*, fruit fly, *D melanogaster*, zebrafish *Danio rerio*

Title	Species	Genetic make-up	Result	Reference(s)
*Yeast*				
*sdh2* KO	*Saccharomyces cerevisiae* (yeast)	KO mutation in S*dh-2* (yeast *SDHB*)	Succinate accumulation; increased ROS production but without evidence of mutagenic DNA damage. Due to excess succinate, showed inhibition of alpha-ketoglutarate-dependent dioxygenases Jlp1 (sulphur metabolism) and Jmj-C domain histone demethylases	[37]
*Copy of human glomus tumor mutation*	*S. cerevisiae*	Conversion of yeast SDH to equivalent of the C191Y human mutation	Increase in ROS production and mitochondrial DNA mutability. Showed that this mutation abolished SDH activity and was truly deleterious	[78]
S*dh-2* (yeast *SDHB*)	*S. cerevisiae*	KO mutation *Sdh-2*	High throughput drug screening. The main upshot was the potential benefit of LDH inhibition	[79]
Copies of human mutations	*S. cerevisiae*	Putting human mutations into yeast SDHx to see if pathogenic	Was correlation in damaging effects between yeast and human equivalent mutations	[80]
*Sdh6* (*SDHAF1*) disruption	*S. cerevisiae*	Disruption of the *SDHAF1* homologue *Sdh6*	SDH deficiency, succinate accumulation, and prevented OXPHOS-dependent growth	[81]
*Sdh5* (*SDHAF2*) disruption	*S. cerevisiae*	Disruption of the *SDHAF2* homologue *Sdh5*	Succinate accumulation; failure in respiratory-dependent growth and reduction in oxygen consumption	[14]
*Sdh8* (*SDHAF4*) disruption	*S. cerevisiae*	Disruption of the *SDHAF4* homologue *Sdh8*	Succinate accumulation, but also maintains approximately 40% of wild type SDH activity	[82]
*Worm*				
Complete *sdhb* KO	*Caenorhabditis elegans*	Complete *sdhb-1* KO	Lethal at L2 larval stage. Sensitivity to hyperoxia. Succinate accumulation, damaged oxygen consumption	[83, 84]
Cell-specific *sdhb-1(RNAi)*	*C. elegans*	Cell-specific *sdhb-1(RNAi)*	Also used DMOG to suppress SDH. Crosses with HIF mutants	[85]
Copy of human mutation in *SDHB*	*C. elegans*	Arg244His point mutant (corresponding to Arg230His in human *SDHB*)	Succinate accumulation, damaged oxygen consumption, increased pyruvate and lactate levels, increased LDH activity. Sterile adults	[84]
*mev-1*	*C. elegans*	G71E in *SDHC*	*mev-1* was discovered in a worm screen for oxygen sensitivity. G71E mutants display oxygen hypersensitivity, decreased lifespan and brood size phenotypes. G71E substitution did not affect succinate-to-fumarate conversion, but led to electron leakage	[86–89]
* sdha-1 *variations	*C. elegans*	Over- and under-expression of *sdha-1*	Loss-of-function mutations in *sdha-1* increases, while *sdha-1* over-expression decreases phosphoenolpyruvate carboxykinase: an inverse correlation between mitochondrial function and the levels of anabolic processes	[90]
*Fruit fly*				
*SDHB* KO	*Drosophila melanogaster* (fruit fly)	*Sdhb-/-*	Defective complex II. Increased hydrogen- peroxide. Hypersensitive to oxygen, progeroid, early death, abnormal flight muscle, mitochondrial abnormalities	[91]
*SDHA* and *SDHB* KOs	*D. melanogaster*	*Sdha -/-* *Sdhb -/-*	Rapamycin treatment improved climbing ability. Rapamycin treatment (inhibits mTor) Extends life of mutants. Rapamycin increased SDH enzymatic activity but no decrease in ROS levels	[92]
Deletion of the *SDHAF4* ortholog *dSdhaf4*	*D. melanogaster*	Deletion of *dSdhaf4*	Neurodegenerative phenotypes, early-adult lethality and sensitivity to oxidative stress. Significant succinate accumulation and almost 90% decrease in SDH activity	[82]
Deletion of the *SDHAF3* ortholog *dSdhaf3*	*D. melanogaster*	Deletion of the *SDHAF3* ortholog *dSdhaf3*	Hypersensitive to oxidative stress; muscular and neuronal dysfunction. Impaired SDH activity and reduced SDHB levels	[93]
*Zebrafish*				
Complete KO of *SDHB*	*Danio rerio*	Complete KO of *sdhb*	Decreased survival; abnormal development (swim bladders). Increased succinate level	[94]

A key advantage of yeast is that it can be haploid. Loss of the *SDHB* (*Sdh2* in yeast) subunit *Sdh2(-)* in a yeast model resulted in increased ROS production but without evidence of mutagenic DNA damage [37]. In the mutant yeast strain, succinate accumulated significantly, which was shown to inhibit two alpha-KG-dependent enzymes: Jlp1, involved in sulfur metabolism and Jhd1, which is a JmjC-domain-containing histone demethylase (JHDMs) enzyme. Smith and colleagues also showed that mammalian JmjC-domain histone demethylases were also sensitive to succinate inhibition *in vitro* and in cultured cells, proposing that any alpha-KG-dependent enzyme might be dysregulated by excess succinate leading to tumorigenesis and or/tumor progression. These findings were underpinned by *in vitro* studies performed by Xiao et al. [33] on human α-KG-dependent enzymes, showing that the structurally similar succinate and fumarate anions can also function as alpha-KG antagonists. As a consequence of inhibiting multiple a-KG-dependent dioxygenases, including the JMJD family KDMs and the TET family of 5mC hydroxylases, excess succinate and fumarate might cause genome-wide alterations in histone and DNA methylation patterns.

The effect of a pathogenic missense mutation (C191Y) in the *SDHB* gene associated with a glomus tumor (i.e. paraganglioma) was examined in yeast. The homologous mutation resulted in abolished SDH activity with increased sensitivity to oxidative stress. In addition, the *sdh2*^*C184Y*^ mutant allele (equivalent to human *SDHB*^*C191Y*^) was not able to rescue the defective OXPHOS phenotype of the *Δsdh2* null, or knockout, mutant. The authors concluded that the C191Y mutation leads to both an increase in ROS production and to mitochondrial DNA mutability [78].

Panizza et al. analyzed a series of missense mutations in *SDHB*, *SDHC* and *SDHD* homologous genes and demonstrated that yeast functions as a good model to validate the pathogenic significance of these mutations, in case they concern conserved amino acid residues in conserved domains [80]. This illustrates how yeast may be used to examine ‘variants of unknown significance’ found in sequencing of the human genome.

In the basic biochemistry, pioneering studies in yeast contributed to the elucidation of the SDH assembly pathway. SDHAF1 was the first SDH assembly factor to be identified. Homozygous *SDHAF1* mutations were detected in patients suffering in infantile leukoencephalopathy, a neurodegenerative disorder similar to Leigh Syndrome [95]. The patients showed elevated succinate levels detectable by *in vivo* proton MR spectroscopy [95]. Disruption of the SDHAF1 yeast homologue *Sdh6* caused SDH deficiency and prevented OXPHOS-dependent growth [81]. Yeast *sdh5*, the homologue of human *SDHAF2*, was first discovered as an uncharacterized mitochondrial protein, which was highly conserved throughout eukaryotes [14]. Deletion of *sdh5* in yeast caused failure in respiratory-dependent growth and reduction in oxygen consumption. Furthermore, Hao et al. identified Sdh1/SDHA as binding partners of Sdh5p, which is required for Sdh1p flavinylation. Subsequently, at least two families with familial paraganglioma have been found to have *SDHAF1* mutations, showing that mutations in SDH assembly factors can recapitulate the phenotype caused by the core subunit mutations [14].

Yeast cells lacking *Sdh8* exhibit accumulation of succinate, but the magnitude of succinate accumulation is much less than that observed in *sdh5Δ* mutants, and *sdh8*Δ mutant yeast maintain approximately 40% of wild type SDH activity [82]. These findings show that Sdh8 is involved in SDH biogenesis, but it is not absolutely required for SDH assembly.

Currently there are no publications associated with human mutations in *SDHAF3* (*sdh7*) or *SDHAF4* (*sdh8*) assembly factor genes.

Thus, yeast has been a very useful and instructive model for basic SDH biochemistry (suggesting new human genes for mutational study), for methylation effects, for metabolism, and for investigation of variants of unknown significance.

2.1.3 *Caenorhabditis elegans* (henceforth, ‘the worm’) is a nematode worm found in the soil. About 1 mm long, it has about 1000 somatic cells whose lineages are understood, just less than half of which form its very primitive nervous system. In as simple an organism as this, ‘neuroendocrine’ cells as such might not be expected, but in fact, the neuron known as ‘RID’ has many features of a neuroendocrine cell [96].

The *C. elegans* mutation originally known as *mev-1* was discovered in a screen for oxygen sensitivity [86]. The name is derived from ‘methyl viologen’, the redox-active weedkiller also known as paraquat. It was later shown that the mutation lay in s*dh3* (*SDHC*) [87]. This homozygous point mutation (G71E in the worm) did not affect the metabolic function of the enzyme (namely to oxidize succinate to fumarate in the TCA cycle), but led to electron leakage, reflecting the role of the *sdh3*/*SDHC* subunit in electron transport [88]. This manifests in oxygen hypersensitivity, decreased lifespan and brood size phenotypes, while a deletional allele of *mev-1* (*mev-(lf)*) is lethal [89]. These phenotypes have been replicated in a mouse model (see in ‘Rodent’ section below), supporting the idea that these processes are evolutionarily conserved [97]. It is of interest that many agricultural pest control agents target SDHx as their mode of action [98], and such agents are being considered for use as human anti-fungal treatments [99].

Recently *mev-1* was used in a proof-of-concept study of miniSOG (‘singlet oxygen generator’)-mediated CALI (‘chromophore-assisted light inactivation’), which is a technique in which spatio-temporally controlled ROS generation is conducted. The use of miniSOG-mediated CALI is a suitable platform for instant inactivation of respiratory chain components [100].

Huang and Lemire [83] observed that different mutations in the *SDHB* gene resulted in ‘superoxide generation and premature aging’. Important to note, that the *C*. *elegans sdhb-1* null mutant’s development arrests midway in development at the L2 larval stage.

Very recently, a copy of the human SDHB Arg230His mutation that presented in a Scottish family has been created in *C. elegans* (Arg244His in the worm) [84]. The findings are at an early stage, but the data show that (i) the enzyme is definitely defective, in that there is an abnormal build-up of succinate; (ii) the worm develops abnormally and is sterile but lives to a later larval stage than the L2-arrested SDHB-deletion worm; and (iii) shows a different pattern of metabolic rearrangement (reminiscent of Warburg effect) compared to the complete SDHB-deletion worm.

Another worm group used convergent genetic and pharmacological approaches to study the effect of SDH deficits on the HIF system in the worm [85]. One effect of HIF activation in the worm is a delay in egg-laying, or ‘egg retention’. To study this, they knocked out SDHB in a subset of neurons, including those thought to be responsible for egg-laying. They also used dimethyloxalylglycine (DMOG), a succinate analogue. Both manoeuvres retarded egg laying; but *not* in HIF-1 knockout worms, showing that the delay in egg-laying is dependent on the HIF system. According to their model, the inhibition of EGL-9 by excess succinate prevents HIF-1 (human HIFα ortholog) hydroxylation; thereby promoting HIF-1 signalling, which eventually leads, among other processes, to the observed retention of eggs.

Lastly, in well-fed worms carrying loss-of-function mutations in *sdha-1*, the expression of phosphoenolpyruvate carboxykinase (PEPCK; *pck-1* and *pck-2*), which converts oxaloacetate to phosphoenolpyruvate and CO_2_, and is part of the gluconeogenic pathway, is increased, while *sdha-1* over-expression has the opposite effect, suggesting an inverse correlation between mitochondrial function and the levels of anabolic (e.g. gluconeogenic, lipogenic) processes [90]. The homozygous *sdha-1* loss-of-function nematodes are viable but show cell non-autonomous behavioral and developmental deficiencies: slower developmental rate from L2 to L3 larval stage; L4 males fail to remodel their anal depressor muscle and are therefore incapable of copulation; show slower movement; and show a slower rate of oxygen consumption. Their mitochondria are smaller and less networked [90].

This illustrates an interesting metabolic re-routing in SDH deficiency, which is also seen in tumors in general [101].

Thus, *C. elegans* can reveal oxidative effects, metabolic effects and developmental abnormalities in SDHx mutants.

2.1.4 The fruit fly *Drosophila melanogaster* is perhaps the longest-used model organism, having been studied (by Morgan and others) since about 1910. Although it has been used very extensively in other biological research, few studies have been done on succinate dehydrogenase. Walker and others [91] were interested in molecular mechanisms that regulate the formation of reactive oxygen species and performed a screen for *Drosophila* mutants that are hypersensitive to high oxygen levels (100%: room air is about 20%). They isolated and characterized an *SDHB* mutant, which survived for only one day in 100% oxygen, while wild-type flies survived for about 7 days. Survival in room air was likewise curtailed. Measurements of hydrogen peroxide showed a 32% increase in the mutants. Mitochondrial structure was abnormal; tests of ageing showed premature ageing in the mutants.

Orthologs of two SDH assembly factors, *dSdhaf3* and *dSdhaf4* were extensively characterized in *Drosophila*. Loss of *SDHAF3/dSdhaf3* in the fruit fly results in impaired SDH activity and reduced SDHB levels, in addition mutant flies are hypersensitive to oxidative stress and display muscular and neuronal dysfunction phenotypes [93]

Deletion of the *SDHAF4* ortholog *dSdhaf4* caused significantly more succinate accumulation and almost 90% decrease in SDH activity, coupled with neurodegenerative phenotypes, early-adult lethality and sensitivity to oxidative stress [82].

Thus, the fruit fly confirms similar observation on ROS seen in *C. elegans* and yeast.

2.1.5 Zebrafish

Recently *Danio rerio* has also shown potential to become an effective model in which to investigate the mechanisms behind PPGL [94]. Homozygous mutant animals with truncated *sdhb* showed shorter lifespans, incompletely or non-inflated swim bladders, defects in energy metabolism and swimming behaviour. In homozygous *sdhb*-mutant larvae, the number and structure of the mitochondria showed no difference from the wild-type, and they observed decreased mitochondrial complex 2 activity and succinate accumulation, as in *SDHB*-associated PPGLs. This novel model offers an opportunity to investigate both HIF activation and alpha-keto-glutarate-dependent dioxygenases, and to screen possible therapeutic targets.

2.1.6 Rodents have been very important in SDH research.
  - *The mouse*

Table 5 shows the mouse and rat models.

**Table 5:** Mouse and rat models of PPGL

Title	Species	Method	Expected adrenal genotype	Result	References
*Mouse models*					
SDHD-ESR TD-SDHD	Mouse	Two floxed *sdhd* mouse models. SDHD-ESR has a tamoxifen-inducible CRE recombinase, while TH-SDHD has a catecholaminergic, tissue specific inducer (tyrosine hydroxylase)	*sdhd -/-*	No tumors, but changes in catecholaminergic cell maturation. The SDHD-ESR mouse was later used to prepare tissues and two cell lines, as described by Millan-Ucles et al. 2014 [74]	[74, 102]
Combined sdhb pten	Mouse	Cross between floxed pten+/- and sdhb +/-mice, targeted by PSA promoter	*sdhb-/- pten -/-*	Tumors occurred but did have residual SDH activity, suggesting that only those with attenuated cre activity, with persisting *sdhb* activity, had actually survived	[103]
Xenograft in NOD-scid (NSG)	Mouse with human xenograft	Viable tissue from human patient tumors injected into subcutaneous tissues of immunodeficient mice	As defined by original patient	Similar to human pathology. Technically difficult and slow process	[104]
iMCC SDHB-KO allograft	Allografts to mouse from iMCC cells	iMCC cells derived from a ‘floxed’ (*sdhb* exon 2) mouse injected into nude mouse	*sdhb-/-*	Useful imaging data obtained by PET-CT	[36, 105, 106]
MTT-*SDHB*^KD^	Mouse	Allograft of MTT cells with *SDHB* knock-down into nude mice	*sdhb*-/-	Definite reduction in both message and SDHB protein SDH, increased succinate. Study of NAD^+^/PARP pathway	[62]
N2a *Sdhb* knockdown allograft	Mouse	N2a (neuroblastoma) cells with *Sdhb* knocked down allografted into mice	*sdhb* KD	Used to study effect of alkaloid piperlongumine on SDH-deficient cells	[65]
RS0 xenograft	Rat-to-mouse xenograft	RS0 *Sdhb*-/- rat tumor cells xenografted into nude mice	*sdhb*-/-	First real *sdhx* KO mouse model; true zellballen architecture	[57]
Rat model					
MENX	Non-*sdhx* pheo model in the rat	Naturally occurring mutant		Carries germline mutation in *Cdkn1b,* coding for cell cycle regulator p27kip1. Pseudohypoxic phenotype, but not *sdhx*-mutated	[107] [108]

It is first important to say that it has proved impossible to generate, by conventional means, a complete knock-out of *sdhx* in a mouse [109], even with organ targeting etc. Sitting as SDH does at the very centre of the metabolism of every living thing on the planet, this is perhaps not too surprising.

The first stab was taken by the Spanish. They produced two floxed *sdhd* mice. The first, SDHD-ESR, had a tamoxifen-inducible promoter, while TH-SDHD was under the control of tyrosine hydroxylase promoter, so that the wild-type alleles could be knocked out (by CRE recombinase) when desired (by tamoxifen) and in the right place (where catecholamines are made) [102]. Regrettably, tumors were not forthcoming, but the SDHD-ESR mouse was used to make tissue for further study and for cell lines [74].

The French group also made two heterozygous mouse models for subsequent mating: a floxed *pten*+/- and a floxed *sdhb* +/- , to try to exploit the propensity for *pten*-mutated mice to develop tumors. When these were crossed, tumors did occur, but regrettably, residual SDH activity was still present, suggesting that the tissue had only survived because the expected CRE-mediated elimination of *sdhb* had been incomplete [103].

Across the ocean, the Tischler lab was able to xenograft viable human tumor tissue into immunodeficient, NSG, mice [104]. This worked, but it is a slow and tricky process, totally dependent on material from human surgery.

The French were able allograft iMCC SDHB-KO cells into NMRI-nu nude mice, to obtain tumors, which allowed a novel form of magnetic resonance imaging aimed at the detection of succinate itself [105].

The Bethesda team used a similar allograft approach, this time using MTT cells with *shdb* knockdown (MTT-*SDHB*^KD^) in nude mice, to investigate the action of olaparib, which targets a DNA repair pathway, in an attempt to exploit this action to sensitise the cells to chemotherapeutic agents, with some success [62]. Again in Bethesda, Bullova and others used N2a neuroblastoma cells (which are neuroendocrine) with *Sdhb* knockdown to allograft nude mice, to study the effect of the alkaloid piperlongumine on high-ROS tumor cells, with promising results [65].

Lastly, Powers and others have used their RS0 (*Sdhb*-/-) rat cell line successfully to xenograft into nude mice [57], making a very promising model. The RS0 xenograft line showed the ‘zellballen’ architecture, did not express SDHB and produced dopamine with low levels of norepinephrine. Metabolic profiling of RS0 xenografts demonstrates succinate and lactate accumulation and transcriptomic analysis shows high expression of HIF2alpha regulatory network components [57].

In the rat, the main mouse line that shows spontaneous formation of PPGL tumors is ‘MENX’, which is not an *Sdhb*-mutated line, but is known to carry a mutation in the cell-cycle regulator p27kip1. However, it does show a pseudohypoxic phenotype [107, 108], a useful feature for students of SDH.

2.1.7 The dog

Curiously the dog is susceptible to a very similar spectrum of malignant diseases as the human, and PPGL is no exception. Keeping dog colonies is prohibitively expensive, and reproduction rates are long in laboratory timescales, but research efforts based on a ‘breed-based genomic approach’ have been begun [110].

Table 6 lists studies on canine PPGL. Canine PPGL is not uncommon in veterinary practice [111]. PCC occurs more often in middle-aged to older dogs. In addition, the incidence of PCC does not depend on breed or gender. Dogs develop metastases in 13–28% of cases, most often in lymph nodes, in the liver, lung, spleen and bones in contrast to humans where the chance of metastases are approximately 10% and concern lymph nodes, liver, lung and bones [112].

**Table 6:** Studies in the dog. The table summarises publications on canine PPGL. These are based on clinical observation by our veterinary colleagues. There have been no attempts to manipulate the dog genome

	Result	References
Collection of 61 cases. No sequencing	Ultrasound is a useful tool in clinical investigation. Metastasis was common	[111]
Six PCs and 2PGLs. Sequenced SDHD and SDHB	One germline *sdhd* mutation was found; many somatic *sdhd* and *sdhb* mutations.	[113]
Measurement of urinary and plasma catechols metanephrines as diagnostic tool	As in humans, the measurement of urinary caecholamines can be instructive	[114]
Clinical review	Some metastasise, some do not	[112]
Molecular study of 50 PPGLs. Immunocytochemistry and *SDHB* + *SDHD* sequencing	Much loss of chromosome 5. Likely pathogenic mutations in SDHB and SDHD found, although no definite germline cases	[115]
Occurrence of both Cushings (ACTH) and pheo is same dog	Case report describing a very unusual combination, which can also occur in human medicine	[116]
Survey of dog tumors from the vet clinic, by immunohistochemistry	A proportion are deficient in SDHA and SDHB	[117]

Diagnosis of PPGLs in dogs can be made by diagnostic imaging (ultrasonography, computerized tomography (CT) and magnetic resonance imaging (MRI)), as well as by biochemical testing [112]. As in human patients, both plasma and urine samples can be tested biochemically. To differentiate pheochromocytoma from other adrenal conditions, measuring urinary normetanephrine:catecholamine ratio seems to be the best option [114]. Urinary and plasma catecholamines and metanephrines in dogs with pheochromocytoma, hypercortisolism, nonadrenal disease and in healthy dogs.

Holt and others identified *SDHB* (pArg38Gln) and *SDHD* (pLys122Arg) mutations in canine PPGL [113]. Korpershoek and others examined 25 PPGL samples by Sanger sequencing and found one case of *SDHB* and 3 cases of *SDHD* mutations [115]. Immunocytochemical and DNA rearrangements were similar to those in human tumors. Abed and others examined 35 PCC samples by immunohistochemistry and the majority of the sections showed SDHx abnormalities: 25 samples lacked SDHB immunoreactivity, whereas 4 samples did not express either SDHA or SDHB [117].

### Model systems: in drug action

2.2.1 Current treatment of PPGL tumors

Aside from surgery, the most frequently used treatments for metastatic PPGL have been chemotherapy and radiation therapy [24]. The most commonly used combination chemotherapy includes cyclophosphamide, vincristine and dacarbazine, known as ‘CVD’ [118]. CVD treatment is commonly used as a first-line therapy for metastatic PPGL, as patients show response to the treatment and have an overall improvement of other symptoms (blood pressure, blood glucose level). However, only about half of the treated patients respond to CVD treatment and after discontinuation of the therapy, a large number of patients show tumor progression [119–121].

Another well-used treatment for metastatic PPGLs is the radionuclide therapy, using radioactively tagged compounds that bind to the tumors. ^131^I-MIBG is one; ^177^Lu-DOTA-SSA is another. Although treatment with ^131^I-MIBG can cause several side-effects including nausea, anorexia, thrombocytopenia, lymphopenia and leukopenia [122, 123], it has been proven that patients who respond to the treatment live longer [124]. As progression of PPGLs can happen rapidly, early treatment after diagnosis has been advised [123, 125, 126].

Studies on *SDHB*-deficient models showed that *SDHB* deletion or knockdown results in an increase of reactive oxygen species. A novel option in PPGL treatment is to further elevate ROS levels in tumor cells, which leads to their apoptosis through DNA damage. Such an approach is to use ascorbic acid to increase the oxidative burden of tumor cells. *SDHB*^*KD*^ allograft-bearing mice were treated with pharmacologic ascorbic acid and showed suppressed tumor growth and longer overall survival [60].

Another way to exert increased oxidative stress on tumor cells is by blocking NRF2, which plays a role in glutathione synthesis. Glutathione is a substrate of glutathione peroxidase, a key enzyme in eliminating ROS. In a recent study Brusatol, an NRF2 inhibitor, resulted in longer survival and suppressed metastatic lesions of PPGL allograft mice. Thus, Brusatol is another promising therapeutic agent in treating PPGLs [127].

As currently used therapies are mainly palliative and not all patients respond, there is a need for new possible treatments concerning PPGL patients. Anti-angiogenic treatment options are under development, such as sunitinib, carbozantinib, axitinib, levantinib and pazopanib [126]. Ongoing PPGL-related clinical trials can be checked on the US Government Clinical Trials website (clinicaltrials.gov) [128, 129].

In parallel with recent chemotherapic and radiotherapic treatments, novel targeted treatment strategies are also under development. Many of these studies are in a preclinical phase, performed on murine and human cell lines and spheroids. The novel agents include BYL719 (a PI3K alpha inhibitor), sunitinib (a receptor tyrosine kinase inhibitor) and everolimus (an mTORC1 inhibitor) [130]. Some of these drugs have been tried in combination. The most promising of these combinations seems to be that of BYL719 and everolimus: the combined effects of these drugs include shrinkage or collapse of tumor cell spheroids, in addition to GSK3 inhibition, cyclinD1/D3 downregulation in PCC cell lines [130]. The HIF1 inhibitor, belzutifan, has recently been shown to be useful in VHL disease [131] and Pacak-Zhuang disease [132] and we understand that clinical trials of this drug are in progress in PPGL.

Pseudohypoxia alters immune system functions; for example, it leads to inactivation of cytotoxic T-cell lymphocytes and increased expression of the immune checkpoint protein programmed death-ligand 1 (PD-L1) and its receptor [133]. PD-L1/PD-1, as important immune checkpoint proteins have been often targeted by novel immune therapies in the last decade. This pathway, among others, functions in the recognition of cancer cell by the immune system. A phase 2 clinical trial of the PD-1 inhibitor pembrolizumab for patients with metastatic pheochromocytoma and paraganglioma is currently ongoing (NCT02721732) [134].

2.2.2 Treatment strategies developed in animal models

Novel treatment strategies are still in need because of the recurrence of PPGLs following treatment. Simple and more complex model systems are frequently used as first options to test new therapeutic drugs in parallel to tumor cell lines and/or mice. We only show some examples of how different model systems have been applied to develop novel treatments or to test the effect of drug candidates.

Yeast SDH mutant cells were also used to conduct high-throughput drug screens by Bancos et al. [79]. These authors screened more than 200,000 compounds to find drugs that are differentially toxic to mutant yeast and identified several inhibitors of alcohol dehydrogenase, which is the yeast equivalent of human lactate dehydrogenase: both regenerate NAD^+^ when TCA cycle function is deranged. Therefore, the authors treated SDH-deficient human HEK293 cells with a lactate dehydrogenase inhibitor (LDHI) and found that they were sensitive to LDHI. Thus, lactate dehydrogenase might be a novel therapeutic target.

Another possible treatment option is the mTOR inhibitor rapamycin, also known as sirolimus (everomilus is a cousin), which was tested in *Drosophila* *sdhA *and *sdhB* mutants. Following rapamycin treatment, the mutant flies lived longer than the non-treated ones. Treated sdhB mutants also showed improved climbing ability and had increased SDH enzymatic activity. However rapamycin treatment did not decrease ROS levels in mutant flies [92].

Vascularisation is an important component of tumor growth and a target for therapy; the NOTCH antagonist delta-like 1 homologue (DLK1) was previously identified as a tumor pericyte-associated antigen in various carcinomas [135], also in renal cell carcinomas [136]. Inhibition of DLK-1 (e.g. vaccination against DLK-1) led to tumor vascular normalization [135].

Verginelli et al. found that Imatinib (Gleevec) that targets endothelial-mural signalling, blocked paraganglioma xenograft formation [8, 9]. In addition, these authors showed that inhibition of DLK1 signaling at the level of PDGFR kinase by imatinib prevented the formation of paraganglioma tumors in nude mice [9], providing a mechanistic rationale for investigating this treatment.

Themozolomide (TMZ), a chemotherapeutic agent combined with the PARP (Poly (ADP-ribose)-polymerase) inhibitor Olaparib (Ola) was tested on a mouse allograft model. In this model athymic nude mice were injected intravenously by *SDHB-*silenced MTT cells. The mechanism behind depends on the fact that the absence of complex II (SDH) in the respiratory chain results in overactivation of complex I, also known as NADH:ubiquinone oxidoreductase, leading to excess NAD^+^ production. PARP, which is known to repair DNA breaks after genotoxic stress, uses the elevated NAD^+^ levels as a cofactor [137, 138]. When the NAD^+^/PARP DNA repair pathway was inhibited by the TMZ/Ola combination, mice survived longer and had less metastatic lesions [62]. Recently, TMZ and Ola treatment option has been translated into an NCI Clinical Trial [139].

## Discussion

There is no definitive therapy for PPGL, whether in the context of *SDH* mutations or otherwise. Model system could be crucial to define both the pathophysiology and the treatability of *SDH*-related cancers.

As Balani and others have pointed out [140], it is difficult to understand the very early stages in the development of tumors. This is especially pertinent in familial conditions such as inherited PPGL, where germline mutations occur in well-understood proteins. Thus, an excellent modelling opportunity is offered, and yet the exact first steps are still unclear. The problem lies in the immense complexity of the interactions, especially in the α-KG-dependent dioxygenases, inhibition of which by succinate has, theoretically at least, thousands of potential consequences in terms of downstream variations in gene expression. The unravelling of such complexity by selective pathway deletion is one of the key ‘tractability advantages’ of simpler organisms in terms of analyzing genetic interactions between different genes. Because such organisms bearing mutated SDH genes have a phenotype, the principle of epistasis analysis can be used to decide whether a mutation in a specific (*SDH*) gene is dependent on mutations in other genes which might otherwise be assumed to code for unrelated cellular events. If the two genes act in the same signaling pathway, the phenotype should change. Thus, crosses between different mutants can be very instructive as novel genetic interactions can be identified, or genes can be positioned in the same pathway, moreover their epistatic (e.g. upstream–downstream) relationships can be determined (e.g. see [141]).

Of course model systems have their disadvantages and in general, there is a trade-off between the simplicity, affordability and rapidity of possible studies in a model system and the closeness of the model system to the human disease. In this review, the easiest and cheapest but most distant model system is the cultured cell, followed by yeast, worms and rodents but the model organism most closely related to the human example is the dog.

One advantage of the simple, invertebrate systems is that phenotypes as a consequence of SDHx dysfunction create signposts to possible therapies. An example is hypersensitivity to high oxygen levels, which is easily shown in yeast, the worm and fruit flies, but oxygen hypersensitivity is hard to measure in more complex systems, although it can be inferred. In addition, genetic manipulation of genes related to reactive oxygen species is possible in yeast and the worm.

Another advantage of simple model systems is that the complete loss of SDH activity in the human tumor is difficult to study because even cultured mammalian cells typically do not survive SDH knock-out. In addition, complete KO of SDH is also embryonic-lethal in mice and rats. But yeast, especially in its haploid form, does survive without SDH and shows the accumulation of succinate and the changes in metabolic directions that are expected [37].

*SDHB* can be also completely knocked out in a non-lethal embryonic manner in *C. elegans* resulting in a L2 larval lethal phenotype which recapitulates metabolic alterations such as succinate accumulation and damaged oxygen consumption as observed in yeast and mammalian cell lines. In addition, both worm and *Drosophila* *SDHB* mutants show sensitivity to oxidative stress. The HIF system is not expressed in yeast, but it is present in *C. elegans* and that has been studied, using delayed egg laying as the measurable outcome. Thus, the field is ready for such studies.

Targeted elimination of genes encoding the 4 subunits of SDH is also possible, especially in mouse and rat via the Cre-LoxP system to establish *SDHB*-modified mice, whose adrenal medullary cells gave rise to the imCC cell line. Separately, heterozygous rats carrying a short SDHB deletion induced by the TALEN method were used to establish xenografts as recently described by Powers et al. This resulted in cell lines RS0 and RS1/2 of chromaffin origin lacking SDHB. The RS0 xenograft is the most successful cell model of *SDHB*-deficient pheochromocytoma, which has the potential to study the HIF system in detail in a mammal.

The dog could be a very useful model, mimicking as it does in its natural existence the occurrence of PPGL in the presence of germline SDHx mutations. Hopefully the ‘breed based genomic approach’ proposed by Davis and Ostrander will bear fruit [110].

The succinate receptor SUCNR1 is widely distributed in nature. It can be found on the surface of many cell types (for example, spleen, liver, retinal ganglion cells), but also expressed on dendritic cells. Succinate is also an inflammatory signal and induces IL-1β expression in the tumor microenvironment, which can lead to a switch of M1 type macrophages, participating actively in anti-tumor immunity. The immunomodulatory role of IL-1β could be best examined in organoid models but thus far is not available. Development of such a novel system would be ideal to study tumor-microenvironment interactions.

There are four outstanding questions in the field of PPGL research: (1) How does the malignant process follow the loss of heterozygosity in the affected cell? (2) Why is it that SDH mutations cause neoplasia in *neuroendocrine* cells in particular? (3) Why is it that some patients with germline SDHB mutations develop metastatic change, while others, even in the same family, do not? (4) How can we improve treatment?

We propose some options provided by model organisms to address the above points.

1. Loss of SDH function primarily leads to succinate accumulation and as we have described above succinate acts as an oncometabolite in different ways and drives tumor progression. A significant event driven by excess succinate is inhibition of alpha KG-dependent enzymes following by alterations in DNA and histone methylation patterns, as seen in yeast. Although the genomic differences between yeast and human make exact parallels difficult, it will be interesting to determine which genomic and histone positions undergo changes in context of methylation patterns. Neither *C. elegans* nor *Drosophila* have been studied in this way, although further epigenetic studies are possible. As epigenetic regulation is almost universal in all the domains of life, any of the model systems offers possibilities here.
2. Although germline *SDHB* mutations are present in every cell, tumor development and progression occur in the majority of cases in neuroendocrine cells. To understand why neuroendocrine cells carrying *SDHB* mutations are in particular prone to neoplasia, one approach is to determine their transcriptomic profile and compare it to that of other cell types. Sequencing of the mRNA pool of isolated, fluorescently labelled cell types at the single cell level is today a feasible approach, for example, in nematode models [142].
3. In metabolic screening studies for mutations in human infants it is often found that many infants remain well despite carrying a potentially lethal genetic disease. A good example is screening for medium chain acyl CoA dehydrogenase deficiency (MCAD) which yields about twice as many screen-positive infants that turn up as compared to the expected number that present with clinical disease. Thus, variability of phenotype is a normal finding and mutant *SDHB* is no exception. Clearly, an adequate network rewiring has occurred in some affected patients.
4. From the therapeutic perspective, we can see how some organisms lend themselves to high-throughput drug screening. Simpler systems, especially yeast, other invertebrate models, such as the worm and fly plus the vertebrate zebrafish allow very large screening projects. Drug candidates can be identified using simple models during screening processes. Simple systems can be also frequently used as first options to test new drugs in parallel to tumor cell lines and/or mice.

In summary, model organisms in this and many other clinical problems permit us to evaluate gene changes found in a patient (or patients) during the increasingly common clinical investigation post genomic sequencing. If a mutation known to be causative is discovered, the position is clear. But often gene changes (or ‘variations’) are identified which are of unclear pathogenic effect and are called ‘variants of unknown significance’. Model systems with easily measurable phenotypic endpoints can be very useful to advance understanding of these variants.

## Abbreviations

α-KG-dependent: Alpha-ketoglutarate-dependent
BPTES: Bis-2-(5-phenylacetamido-1,3,4-thiadiazol-2-yl) ethyl sulfide
BYL719: A PI3 kinase inhibitor
CALI: Chromophore-assisted light inactivation
*C. elegans*: *Caenorhabditis elegans*
c-Myc: Derived from ‘myelocytomatosis’
Cre-loxP: ‘Causes recombination’- ‘locus of X-over P1’
CRISPR: Clustered regularly interspaced short palindromic repeats
CT: Computerised tomography
DLK1: Delta-like homologue 1
EGLN: Egl nine
FAD: Flavin adenine dinucleotide
GLS-1: Glutaminase 1
GPR91: G protein-coupled receptor number 91
GSK3: Glycogen synthase kinase, 3
HCT116: Human colon cancer cell line
HEK293: Human embryonic kidney 293
HeLa: Henrietta lacks
HIF: Hypoxia-inducible factor
hTERT: Human telomerase reverse transcriptase
IL-1β: Interleukin 1 beta
iMCC: Immortalized mouse chromaffin cells
JHDM: JmjC-domain-containing histone demethylase
Jmjd: Jumonji domain-containing
KDM: Histone lysine demethylase
KD: Knock down
KO: Knock out
Lu-DOTA-SSA: Lutetium 2,2′,2″,2‴-(1,4,7,10-tetraazacyclododecane-1,4,7,10-tetrayl)tetraacetic acid somatostatin
MCAD: Medium chain acyl CoA dehydrogenase deficiency
MPC: Mouse pheochromocytoma
MENX: Multiple endocrine neoplasia-X
*Mev*: Methyl violet
MIBG: *Meta*-Iodobenzylguanidine
MRI: Magnetic resonance imaging
mTORC: Mammalian (or ‘mechanistic’) target of rapamycin complex
MTT: Mouse tumor cells
NAD: Nicotinamide adenine dinucleotide
Nf1: Neurofibromin 1
NOD: Non-obese diabetic
NOTCH: (Gene/protein name derived from ‘notched’ appearance of fruit fly wings)
NSG: NOD scid gamma
OXPHOS: Oxidative phosphorylation
PARP: Poly (ADP-ribose)-polymerase
PC12: Pheochromocytoma 12
PCC: Pheochromocytoma
PDX: Patient-derived xenograft
PEPCK: Phosphoenolpyruvate carboxykinase
PET-CT: Positron emission tomography-computerised tomography
PHDn: Prolyl hydroxylase n
PI3: Phosphatidyl inositol
PPGL: Pheochromocytoma paraganglioma
ROS: Reactive oxygen species
RS0: (Cell line designation)
RS1/2: (Cell line designation)
SDH: Succinate dehydrogenase
SDHx: Denoting any of the four subunits, SDHA, B, C or D, of the complete enzyme
SDHAF1: Succinate dehydrogenase assembly factor 1
*S. cerevisiae*: *Saccharomyces* *cerevisiae*
Scid: Severe combined immune deficiency
STAT3: Signal transducer and activator of transcription 3
SUCNR1: Succinate receptor 1
TALEN: Transcription activator-like effector nuclease
TCA: Tricarboxylic acid (cycle)
TET: Ten-eleven translocation
TMZ: Themozolomide
VHL: Von Hippel Lindau
